# OMD Curation Toolkit: a workflow for in-house curation of public omics datasets

**DOI:** 10.1186/s12859-024-05803-9

**Published:** 2024-05-09

**Authors:** Samuel Piquer-Esteban, Vicente Arnau, Wladimiro Diaz, Andrés Moya

**Affiliations:** 1 Institute for Integrative Systems Biology (I2SysBio), University of Valencia and Spanish National Research Council, Valencia, Spain; 2 Area of Genomics and Health, Foundation for the Promotion of Sanitary and Biomedical Research of Valencia Region (FISABIO-Public Health), Valencia, Spain; 3 Biomedical Research Networking Centre for Epidemiology and Public Health (CIBEResp), Madrid, Spain

**Keywords:** Omics data, Curation tools, Data integration, ENA, Metadata, Sequencing data

## Abstract

**Background:**

Major advances in sequencing technologies and the sharing of data and metadata in science have resulted in a wealth of publicly available datasets. However, working with and especially curating public omics datasets remains challenging despite these efforts. While a growing number of initiatives aim to re-use previous results, these present limitations that often lead to the need for further in-house curation and processing.

**Results:**

Here, we present the Omics Dataset Curation Toolkit (OMD Curation Toolkit), a python3 package designed to accompany and guide the researcher during the curation process of metadata and fastq files of public omics datasets. This workflow provides a standardized framework with multiple capabilities (collection, control check, treatment and integration) to facilitate the arduous task of curating public sequencing data projects. While centered on the European Nucleotide Archive (ENA), the majority of the provided tools are generic and can be used to curate datasets from different sources.

**Conclusions:**

Thus, it offers valuable tools for the in-house curation previously needed to re-use public omics data. Due to its workflow structure and capabilities, it can be easily used and benefit investigators in developing novel omics meta-analyses based on sequencing data.

**Supplementary Information:**

The online version contains supplementary material available at 10.1186/s12859-024-05803-9.

## Background

Significant advances in sequencing technologies have resulted in many available omics datasets (metataxonomics, metagenomics, metatranscriptomics, RNA Seq, etc.) in public databases. However, attempting to reutilize public datasets effectively is challenging [1, 2]. Issues usually include: (i) incomplete, scattered, or even absent metadata; (ii) the need for further treatment of sequencing files (e.g., appropriate concatenation of files to obtain the final sample's files); and (iii) uneven data processing and quality control. As a result, a careful curation process is required before any utilization.

While there are initiatives that aim to support the re-use of previous results [3–5], some of them, even with a particular focus on metadata curation [6–10], often present some limitations that usually lead to the need for further in-house curation and processing, such as: (i) datasets of interest may not be present if they are too recent; (ii) the need to use alternative processing pipelines (e.g., specialized virome pipelines instead of the standard prokaryotic ones in metagenomic data) or more recent available versions; and (iii) variable selection might result in the omission of other available metadata variables. Furthermore, whereas it is not difficult to find standard operating procedures (SOPs) and pipelines [11–14] that can perform the latter data reprocessing and even meta-analysis, unified tools for the crucial previous curation process of metadata and fastq files are needed. While there are tools associated with the main sequencing databases [5, 15–18], they are often database-specific, collection-focused, and in some cases, scattered across different tools (see Supplementary Table 1 in Additional file 1). For this purpose, we have developed the Omics Dataset Curation Toolkit (OMD Curation Toolkit).

## Implementation

**Fig. 1:**
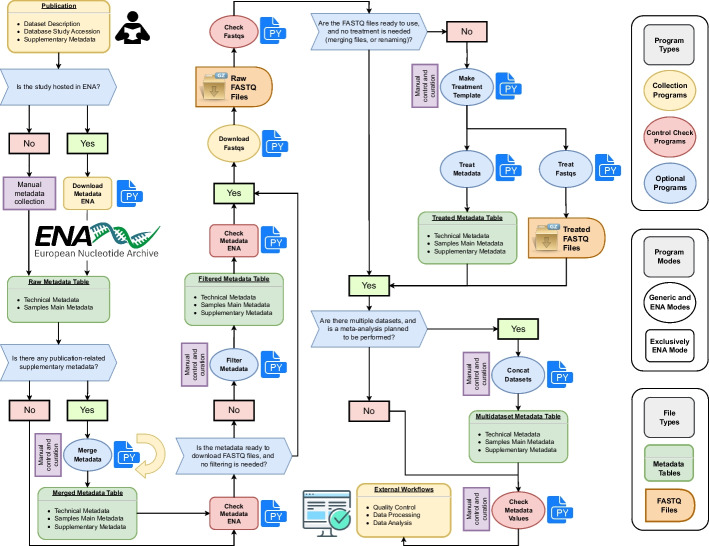
**Overview of the OMD Curation Toolkit workflow**. Firstly, the original publication is reviewed to obtain the necessary context, supplementary metadata, and study accessions. When working with datasets hosted in ENA[15] or other related databases, the Download Metadata ENA program can download all the metadata available in ENA (alternatively, the user would need to do this metadata collection process manually). If additional metadata is available, this can be incorporated using the Merge Metadata program. Then, the Check Metadata ENA program can perform a metadata screening if the dataset is ENA-related. The Filter Metadata program can select the metadata of interest before taking further steps. Then, once the samples and associated files of interest are chosen, the Download Fastqs program can be used to download the fastq files, and the Check Fastqs program to carry out a complete screening of the downloaded fastq files. The three treatment programs can also be used if further treatment is needed (renaming or merging of fastq files and combining the associated metadata). Furthermore, if multiple datasets are curated, they can be securely integrated using the Concat Datasets program. Finally, the Check Metadata Values program can be used to check that the curated metadata variables are within the allowed parameters

OMD Curation Toolkit is a suite of command-line tools specially designed to download and curate metadata and fastq files of public omics datasets hosted in the European Nucleotide Archive (ENA)[15]. As a member of the International Nucleotide Sequence Database Collaboration (INSDC), ENA shares information with other major sequencing databases of interest, including the Sequence Read Archive (SRA) and the DNA Data Bank of Japan (DDBJ), allowing access to a large amount and diversity of omics data. Nonetheless, the majority of the available package tools are generic and can be used to curate datasets from different sources, providing additional versatility. Implemented entirely as an open-source Python3 package (> = 3.10), its core functionalities depend mainly on standard Python libraries. In contrast, third-party open-source Python libraries carry out other aesthetics options (termcolor and tabulate libraries) and functionalities (pandas, mg-toolkit, and parfive libraries). OMD Curation Toolkit is implemented in a workflow fashion in which each program corresponds to a different curation step (Fig. 1). The source code is publicly available as a GitHub repository (https://github.com/tbcgit/omdctk) and also via PyPi (https://pypi.org/project/omdctk/). The GitHub repository has a complete wiki documentation with specific pages for each program (explaining its operation, parameters, and use examples), installation options, considerations for curating metadata in public datasets, and a complete case example of dataset curation for both ENA and external datasets (https://github.com/tbcgit/omdctk/wiki).

## Results and discussion

### Collection programs

These programs correspond to the collection workflow steps that allow obtaining the metadata and fastq files associated with the dataset of interest.

- **Download Metadata ENA** allows downloading the metadata associated with an ENA study project by collecting the information available in the ENA Browser and the metadata related to the project's samples using the mg-toolkit package (https://pypi.org/project/mg-toolkit/). Alternatively, the user would need to manually do this metadata collection process using other package tools or external tools, depending on the source database in which the dataset is hosted.
- **Download Fastqs** allows the downloading of the associated fastq files from an ENA study project based on the information in the provided metadata table using the parfive package (https://pypi.org/project/parfive/). Alternatively, a generic mode that accepts URLs in a text file is also available to re-launch URLs from error reports that could not be downloaded in previous attempts or to download fastqs, metadata tables, or other files from different sources.

### Control check programs

These programs correspond to the control points of the workflow that support the checking, verification, and interpretation of the obtained files of the dataset. They are designed to analyze the various files and provide help messages to assist the researcher during the curation process, indicating what could be the reasons for concern (if any) and what should be done.

- **Check Metadata ENA** enables the principal analysis and checks related to the ENA metadata tables generated at different workflow points. Relevant statistics for run accessions, as well as samples, will be calculated, including the number of run accessions, number of samples, appearances per organism (scientific name and tax id), sequencing platform (instrument model and platform), library layout (PAIRED or SINGLE files) and data type (library strategy and source). It also performs a grouping of samples by the number of associated runs. Likewise, relevant checks are performed for run accessions, including comparing the library layout with the number of available fastqs, the availability of the original uploaded fastqs, and the presence of duplicated names in these files. It also conducts checks per sample, including comparing the number of runs and samples, comparing the samples of the provided sample columns, and checking the presence of multiple matches per sample for an organism, sequencing platform, library layout, and data type.
- **Check Fastqs** enables some checks of interest on the downloaded fastq files, including confirming that all expected files from the metadata or manifest tables exist in the download directory, detecting fastq files absent in the metadata or manifest tables, and checking the presence of multiple matches for the fastq files in the metadata or manifest tables. By default, the program works with the ENA metadata, but it also has a generic mode based on a manifest table file. A comparison between the manifest and metadata tables will also be performed if the generic mode is selected. Furthermore, it can also check the integrity of the files by calculating MD5 sum checks.
- **Check Metadata Values** performs a series of analyses and checks on the values of the curated metadata table based on the information in the provided variables dictionary file. For each of the provided variables a series of analyses will be performed, including checking the presence of the variable in the curated metadata table according to its requiredness nature (depending on whether is indicated as a required or an optional variable), check the type of the variable based on its class type (verify that these are strings or booleans if indicated as a character variable, or numerical values if indicated as a numeric variable), checking uniqueness within variable (depending on whether is indicated as unique or nonunique values), check the presence of multiple matches when comparing the uniqueness between the variable and a set of variables of interest (especially useful for detecting inconsistencies related to duplicates, such as the presence of identical individual identifiers between different datasets), and check that the values of the variable are within the allowed parameters provided with different analyses available (any, subset, wholeset, or numeric range).

### Optional curation programs

These programs correspond to extra workflow steps that provide further functionalities for curating and integrating metadata and fastq files.

- **Merge Metadata** provides different options for merging metadata tables to combine metadata from various sources. By default, a left join will be carried out, combining a main metadata table (usually the ENA metadata obtained from the Download Metadata ENA program) and an extra metadata table (usually the publication's metadata), taking the former as the left reference. Nevertheless, the program is generalist, provides alternative merging options, and can be used to merge metadata from external projects. After merging, the values of the two provided merge columns will be analyzed by performing an intersection to check for non-common unique values.
- **Filter Metadata** allows different filtering operations to be performed sequentially on the previously generated metadata table based on the provided filtering information. This program allows the application of various categorical and numerical filters and supports the treatment of unavailable (NA) values.
- Three treatment programs can also be used together for further fastq and metadata treatment. **Make Treatment Template** allows to generate a raw treatment template using the information in the metadata or manifest tables and the downloaded fastq files. The resulting template file must be further curated so subsequent workflow programs can use it (**Treat Fastqs** and **Treat Metadata**). These programs can be used for the extra treatment of fastq files and the combination per sample of their associated metadata with three possible treatment operations (copy, rename, and merge). Furthermore, in the case of Treat Metadata, if multiple different values are found per sample, the program will generate a warning report to detect possible metadata inconsistencies.
- **Concat Datasets** combines the curated final metadata tables of the different datasets of interest. The information in the provided variables dictionary file will be used to concatenate the rows of the different metadata tables found. It is beneficial if we are interested in conducting a meta-study with multiple datasets.

## Conclusions

Despite the growing number of sequencing projects in public databases, working with public omics datasets remains challenging. Even with initiatives aimed at re-using previous results, it is easy to find limitations that often lead to the need for further in-house curation and processing beyond the mere collection of datasets. The OMD Curation Toolkit provides a standardized framework to facilitate, accompany and guide the researcher during this manual curation process. With its workflow structure and capabilities (collection, control check, treatment, and integration), this toolkit can be easily incorporated into any laboratory's SOPs, boosting the incorporation of public datasets and the development of novel omics meta-analyses.

## Availability and requirements

Project name: OMD Curation Toolkit.

Project home page: https://github.com/tbcgit/omdctk

Operating system(s): Platform independent.

Programming language: Python3.

Other requirements: Python3(> = 3.10).

License: MIT.

Any restrictions to use by non-academics: none.

### Supplementary Information

**Additional file 1. Supplementary Table 1.** Comparison of different omics data tools. 

## Data Availability

All data and materials needed for installing and running the workflow, as well as a complete case example of both ENA and external dataset curation, are included on the OMD Curation Toolkit GitHub site at https://github.com/tbcgit/omdctk. The complete case of dataset curation can be followed step by step at the tutorial example (https://github.com/tbcgit/omdctk/wiki/Tutorial-Full-Example).

## Abbreviations

ENA: European nucleotide archive
SRA: Sequence read archive
DDBJ: DNA data bank of Japan
INSDC: International nucleotide sequence database collaboration
NA: Not available
OMD: Omics dataset
SOP: Standard operating procedure
